# Biofilm formation of two genetically diverse *Staphylococcus aureus* isolates under beta-lactam antibiotics

**DOI:** 10.3389/fmicb.2023.1139753

**Published:** 2023-03-06

**Authors:** Jinglong Liang, Teng Yi Huang, Yuzhu Mao, Xuejie Li

**Affiliations:** ^1^College of Light Industry and Food Technology, Zhongkai University of Agriculture and Engineering, Guangzhou, China; ^2^Guangdong Provincial Key Laboratory of Lingnan Specialty Food Science and Technology, Zhongkai University of Agriculture and Engineering, Guangzhou, China; ^3^Key Laboratory of Green Processing and Intelligent Manufacturing of Lingnan Specialty Food, Ministry of Agriculture, Zhongkai University of Agriculture and Engineering, Guangzhou, China; ^4^Department of Diagnostics, Second Affiliated Hospital of Shantou University Medical College, Shantou, China; ^5^Department of Civil and Environmental Engineering, University of Maryland, College Park, MD, United States; ^6^School of Food Science and Engineering, Guangdong Province Key Laboratory for Green Processing of Natural Products and Product Safety, Engineering Research Center of Starch and Vegetable Protein Processing Ministry of Education, South China University of Technology, Guangzhou, China; ^7^Research Institute for Food Nutrition and Human Health, Guangzhou, China

**Keywords:** *Staphylococcus aureus*, biofilm, sub-MICs, antibiotics, SCC*mec*

## Abstract

**Purpose:**

Our aim was to evaluate the biofilm formation of 2 genetically diverse *Staphylococcus aureus* isolates, 10379 and 121940, under different concentrations of beta-lactam antibiotics on biomass content and biofilm viability.

**Methods:**

Biofilm formation and methicillin resistance genes were tested using PCR and multiplex PCR. PCR was combined with bioinformatics analysis to detect multilocal sequence typing (MLST) and SCC*mec* types, to study the genetical correlation between the tested strains. Then, the crystal violet (CV) test and XTT were used to detect biomass content and biofilm activity. Antibiotic susceptibility was tested using a broth dilution method. According to their specific MIC, different concentrations of beta-lactam antibiotics were used to study its effect on biomass content and biofilm viability.

**Results:**

Strain 10379 carried the *icaD*, *icaBC*, and MRSA genes, not the *icaA*, *atl*, *app*, and *agr* genes, and MLST and SCC*mec* typing was ST45 and IV, respectively. Strain 121940 carried the *icaA*, *icaD*, *icaBC*, *atl*, and *agr* genes, not the *aap* gene, and MLST and SCC*mec* typed as ST546 and IV, respectively. This suggested that strains 10379 and 121940 were genotypically very different. Two *S. aureus* isolates, 10379 and 121940, showed resistance to beta-lactam antibiotics, penicillin, ampicillin, meropenem, streptomycin and kanamycin, some of which promoted the formation of biofilm and biofilm viability at low concentrations.

**Conclusion:**

Despite the large differences in the genetic background of *S. aureus* 10379 and 121940, some sub-inhibitory concentrations of beta-lactam antibiotics are able to promote biomass and biofilm viability of both two isolates.

## 1. Introduction

*Staphylococcus aureus* (*S. aureus*) is a common food borne pathogen, responsible for a large variety of food borne infections and diseases, particularly in food poisoning (by causing staphylococcal food poisoning) and food spoilage (Xu et al., 2016b; Miao et al., 2017, 2019; Idrees et al., 2021). This pathogen is often found in the air, soil, water, and various food animals such as pigs, sheep, and cows (Argudín et al., 2010), and thus can be commonly carried by food and ultimately endanger human health (Xu et al., 2012; Jiang et al., 2021). The US Centre for Disease Control reports that *S. aureus*, just behind *Escherichia coli*, is the second most prevalent pathogenic bacteria. Methicillin-resistant *S. aureus* (MRSA) has been considered to be a leading pathogen closely relevant to human beings, firstly identified as hospital associated (HA-MRSA), then community associated (CA-MRSA) (Xu et al., 2011; Deng et al., 2015b; Shrestha et al., 2019, 2021). In the recent decade, however, a large number of studies have reported MRSA in animals, and subsequently in animal meat and raw food samples. Known as livestock associated MRSA (LA-MRSA) and belonging to ST 398/CC 398, LA-MRSA has been reported to be more and more prevalent (Silva et al., 2022; Gao et al., 2023). One distinctive characteristic of MRSA is the carriage of a mobile element SCC*mec* (Staphylococcal chromosomal cassettes *mec*) genomic island, containing a critical set of gene sequences in MRSA (Hanssen and Ericson Sollid, 2006; Turlej et al., 2011; Ito et al., 2014; Yang et al., 2015; Liu et al., 2016a; Uehara, 2022). If the study of SCC*mec* genomic islands in *S. aureus* is to be achieved, it is necessary to detect MRSA in *S. aureus*. MRSA is mainly found in *S. aureus* strains carrying the sequence of the *mecA* gene, responsible for the synthesis of the PBP2a (Penicillin Binding Protein 2a), which is resistant to beta-lactam antibiotics with low affinity (Ito et al., 2009). The strain is defined as MRSA when the test bacterium is detected as carrying the *mecA* gene or the PBP2a protein (Stenholm et al., 2011). In addition to SCC*mec*, antimicrobial resistance mechanisms, such as class 1 integrons, had also been occasionally reported in MRSA (Xu et al., 2007, 2008a,b, 2011). Collectively, MRSA is a superbug and being reported to be more and more prevalent in animal foods. In addition, overuse of antibiotics in veterinary remains a leading concern, which has posed an important concern as the consequent correlation between the residual antibiotics and MRSA.

*Staphylococcus aureus* is capable of forming biofilm, a protein adsorption layer formed by hydrophobic proteins or polysaccharides adhering to the surface of biotics or non-biotics (Archer et al., 2011; Park and Seo, 2019). Polysaccharides, proteins, and nucleotides are the main components of the extracellular polymeric substances (EPS) of biofilm, which make up approximately 90% of the dry weight of the biofilm and constitute the immediate environment in which the microorganisms live (Kaplan et al., 2012; Idrees et al., 2021). Biofilm formation consists of two main stages, primary bacterial attachment and interbacterial adherent aggregation (Moormeier et al., 2014). The initiation of the biofilm in *S. aureus* is regulated by the autolysin (*atl*) gene (Biswas et al., 2006; Houston et al., 2011). Autolysin promotes biofilm adhesion on the one hand and has some autolytic enzymatic activity on the other, suggesting that AtlA may have a bidirectional function in the formation of biofilm (Biswas et al., 2006; Chen et al., 2013). The latter stage is associated with polysaccharide intercellular adhesin (PIA), which is regulated and modified by the intercellular adhesion (*ica*) locus, including the *icaA*, *icaD*, and *icaBC* genes (Cramton et al., 1999; Arciola et al., 2001; Götz, 2002; Idrees et al., 2021; Mohammadi Mollaahmadi et al., 2021). The formation of the extracellular aggregation-associated protein AAP is encoded by the *aap* gene (Hussain et al., 1997), which is associated with the aggregation of the bacteriophage after biofilm formation (Patel et al., 2012). *S. aureus* has also been well studied for its quorum sensing system, mostly the *agr* system. The spreading and migration of the biofilm during maturation is regulated by the *agr* gene, which is responsible for the population sensing system of staphylococci and is critically involved in biofilm formation (Vuong et al., 2000; Boles and Horswill, 2008; Dotto et al., 2021). In addition to biofilm formation, agr system also plays a critical role in interspecies polymicrobial interaction (Xu et al., 2019, 2021; Liu et al., 2021, 2022), as *S. aureus* has also been commonly studied in polymicrobial interaction with Pseudomonas aeruginosa, Candida albicans, etc. Biofilm formation considerably differs in species or even isolates, and how to determine the biofilm ability still remains controversial. A large number of studies had employed CFU or CV. However, formation of viable but non-culturable (VBNC) state would yield false negative results when CFU is applied, as VBNC cells are non-culturable on medium plates (Liu et al., 2017c). VBNC state has been studied in various food pathogens and spoilage microorganisms (Liu et al., 2018b; Xu et al., 2020), and VBNC cells could maintain its ability in food poisoning and spoilage (Liu et al., 2017a,b), which has posed a significant concern for food safety. In a previous study (Xu et al., 2016a), CV and XTT have been employed to test the biofilm formation in a large scale of *S. aureus* strains, and it was found out that, indiscrepancy was found between the CV and XTT results, as the two methods study the biomass and viability within biofilms, respectively. Therefore, in this study, different from most studies, both CV and XTT are employed.

Antibiotics are the most commonly used treatment for *S. aureus* (Pantosti et al., 2007). In the 1950s, antibiotics began to be used as a feed additive, which gradually made animal husbandry one of the most used areas for antibiotics (Chambers and DeLeo, 2009). Approximately 300,000 tonnes of antibiotics are used worldwide each year, of which approximately 80% are veterinary antibiotics (Li, 2017; Chowdhury et al., 2021). Commonly used antibiotics in feed include tetracycline, erythromycin, penicillin, and lincomycin (Moyane et al., 2013). The misuse of antibiotics in animal husbandry can lead to residues in the environment, antibiotic residues in food, and bacterial resistance (Soulsby, 2007; Marshall and Levy, 2011). The content of antibiotic residues in food is relatively low compared to the level of antibiotics used in livestock, typically 0.01–100 μg/kg, but have a significant impact on microorganisms. The US Food and Drug Administration and the Centers for Disease Control have jointly stated that there is a link between the long-term consumption of meat containing antibiotic residues and the loss of efficacy of medical antibiotics (Rule et al., 2008; Chee-Sanford et al., 2009). The residues of antibiotics in food can also affect other aspects of bacteria, such as the ability to form biofilms. Wu et al. (2014) found that beta-lactam antibiotics could promote biofilm formation by upregulating carbohydrate metabolism in *Haemophilus influenzae*. Also, effect of ampicillin on *S. aureus* had been reported (Lee et al., 2013; Liu et al., 2018c). Rachid et al. (2000) found that sublethal concentrations of tetracycline could promote biofilm formation in staphylococci, mainly due to the promotion of expression of *ica*.

Therefore, considering different beta-lactamase antibiotics, biomass and viability in biofilms, as well as different genetical diversity within strains, the biofilm formation remains unclear. In this study, we aimed to investigate the inhibitory effect of beta-lactam antibiotics on *S. aureus* 10379 and 121940, and the effect of sub-inhibitory antibiotics on the biomass content and biofilm viability of the two strains of *S. aureus*.

## 2. Materials and methods

### 2.1. Strains and culture conditions

*Staphylococcus aureus* 10379 and 121940 were initially stored at –80°C in 20% glycerine (Liu et al., 2023). The frozen strains were taken from the –80°C refrigerator and thawed at room temperature. 20 μl of the glycerol-preserved bacterial solution was inoculated to 2 ml of TSB (Tryptone Soy Broth) medium (Huan Kai Microbial Technology Company, Guangzhou) and shaken at 37°C for 12 h at 200 rpm. The *S. aureus* culture was dipped with an inoculating loop, scratched on TSA (Tryptone Soy Agar) medium then put in a 37°C incubator for 12 h. One colony of *S. aureus* from the plates was picked, transferred to TSB medium and shaken for 8 h at 37°C to obtain logarithmic growth stage *S. aureus*. The OD600 value of the activated broth was measured using a UV spectrophotometer and diluted to 0.001. This dilution was used for subsequent antibiotic susceptibility testing.

### 2.2. Genomic DNA extraction and primers design

One milliliter of bacterial broth in the logarithmic growth phase was placed in a 1.5 ml sterile EP tube and centrifuged at 12000 r/min for 1 min to remove the liquid medium. 180 μl of lysozyme was filled to the bacterial precipitate and then mixed well. The EP tube was placed in a 37°C water bath for 1 h. After the water bath, 4 μl of RNase A (100 mg/ml) was loaded into the EP tube and then left the tube at 25°C for 5 min. 220 μl of lysate MS was added to the EP tube and shaken to mix. Then the tube was placed in a warm bath at 70°C for 15 min. 220 μl of anhydrous ethanol was added to the EP tube and mixed well by inverting, and briefly centrifuged to remove the beads from the inside of the cap. DNA Rapid Extraction Kits were used to purify the whole genome DNA (Guangzhou Dongsheng Biotechnology Co., Guangdong, China) (Wang et al., 2018). The software Primer Premier 5 was used to design the primers, which were synthesized by Invitrogen, Thermo Fisher Scientific. All primers that were used for this research were shown in Supplementary Table S1. The PCR was operated according to the kit instructions (Li et al., 2019, 2021).

### 2.3. Genotyping of *Staphylococcus aureus* strains

Two *S. aureus* strains were subjected to a number of genotyping assays, firstly by SCC*mec* typing. Bioinformatics analysis of SCC*mec* was performed based on the results of ccr complex and *mec* complex typing. By applying for the IWG-SCC program in the SCC database by going to http://www.sccmec.org and following the prompts to enter the ccr complex and *mec* complex information respectively, information such as genomic island SCC*mec* types and subtypes were finally obtained. Then, MLST and spa typing were also performed accordingly (Goudarzi et al., 2017), followed by detection of a few biofilm associated genes, including *atl*, *ica* operon, and *agr* (Cramton et al., 1999).

### 2.4. Antimicrobial susceptibility testing and MIC determination

The microbial broth dilution method, one of the standard experimental methods commonly used in clinical testing to determine the MIC (minimum inhibitory concentration), was applied to test the drug resistance of *S. aureus*. The storage solutions of penicillin, ampicillin, meropenem, streptomycin, kanamycin, and gentamycin were prepared in H_2_O or DMSO at a concentration of 1,280 μg/ml, except for kanamycin, for which the storage solution is 4,096 μg/ml. Then the storage solutions were diluted with TSB medium in a gradient to 10 different concentrations. The diluted bacterial solution in 2.1 was mixed 1:1 with TSB medium containing different ampicillin concentrations and added to sterile clear 96-well cell culture plates with 200 μl per well, using the bacterial suspension without ampicillin and fresh TSB medium as controls, and three parallel groups were set up for each group. At the end of incubation, MIC was identified to be the minimum concentration clarified in the wells (Fraser-Pitt et al., 2016).

### 2.5. Antibiotic-stressed biofilm forming in *Staphylococcus aureus*

The storage solutions of beta-lactam antibiotics were diluted to 10 concentrations of 1/128, 1/64, 1/32, 1/16, 1/8, 1/4, 1/2, 1, 2, and 4 MIC using TSB medium. Hundred μl of the diluted working solution was then added to a sterile 96-well plate. Each well was filled with 100 μl of the diluted bacterial solution in 2.1 and cultured at 37°C for 48 h. The total amount of biomass and biofilm activity was measured at five-time points, 0, 8, 16, 24, and 48 h, respectively (Mlynek et al., 2016).

### 2.6. Determination of total biomass content

Crystal Violet assay (CV) was used to assess the total amount of biomass. After the 96-well plate had been incubated, the suspension was decanted, and each well was rinsed three times with sterile saline. A volume of 200 μl of saline was added each time to remove the planktonic bacteria. Then each well was added with 200 μl of sterile crystal violet solution at 0.01% and stained for 15 min. After staining, the wells were decanted and washed twice with sterile water, each time adding 200 μl of sterile water per well. Afterwards, each well was loaded with 200 μl of 95% ethanol and the stain was removed over 15 min. Hundred and twenty-five μl of the eluate was placed in a new ELISA plate (Thermo Fisher Scientific) and the optical density at 540 nm was measured. In this part of the experiment, each sample was repeated three times.

### 2.7. Determination of biofilm viability

This experiment uses Promega’s MTS solution, which is similar in principle to XTT but simpler, requiring only a pre-dilution, to assess the viability of the *S. aureus* biofilm. After the 96-well plate had been incubated, the suspension was decanted and each well was then treated with sterile PBS buffer three times. Each time 200 μl was added to remove the planktonic bacteria. Two hundred μl of MTS working solution was loaded to each well of the 96-well plate which was wrapped in tin foil to protect them from light and left to stand for 2.5 h in a 37°C incubator. Hundred and twenty-five μl of the stained solution was loaded into a new ELISA plate (Thermo Fisher Scientific) and the absorbance values were tested at 490 nm. For this part of the test, each sample was repeated three times. The figures were prepared using Microsoft Excel software.

### 2.8. Statistical methods

One-way analysis of variance (ANOVA) was used to determine if the difference was statistically significant. “*” means *p* < 0.05; “**” means *p* < 0.01; “***” means *p* < 0.001.

## 3. Results and discussion

### 3.1. Genotyping of *Staphylococcus aureus* biofilm-related genes

To investigate whether the two *S. aureus* isolates carry the *atl* gene, the whole genomes of them were verified by PCR. Table 1 shows that strain 10379 did not carry the *atl* gene, while strain 121940 carried the *atl* gene. The PCR amplification products of the *icaA*, *icaD*, and *icaBC* operons were 188, 198, and 1,188 bp, respectively. Strain 10379 did not carry the *icaA* operon, while strain 121940 carried the *icaA*, *icaD*, and *icaBC* operons. In addition, strain 10379 did not carry the *agr* gene, while strain 121940 carried the *agr* gene.

**Table 1:** The genetic background of *Staphylococcus aureus* isolates.

Strains	MRSA	Genes associated with biofilm synthesis						MLST	Mec complex	Ccr complex	SCC*mec*
		*icaA*	*icaD*	*icaBC*	*atl*	*aap*	*agr*				
10379	+	−	+	+	−	−	−	ST45	B	2	IV
121940	+	+	+	+	+	−	+	ST546	B	2	IV

“+” means the gene is present in the strain; “–” means the gene is not present in the strain.

### 3.2. Correlation of genomic island SCC*mec* with biofilm genotypes

In addition, the types of SCC*mec* of strains 10379 and 121940 were analyzed. According to the typing results of the Ccr and Mec complex (Ito et al., 2009), the SCC*mec* of two strains belonged to type IV (Table 1; Shore et al., 2010; Albrecht et al., 2011). For Multilocus sequence typing (MLST) testing of strains 10379 and 121940, 10379 strains belonged to ST45 and 121940 belonged to ST546. As summarized, the genetical background of 10379 and 121940 is highly diverse, according to their ST types and the carriage of biofilm associated genes.

### 3.3. Determination of biofilm formation ability

**Figure 1:**
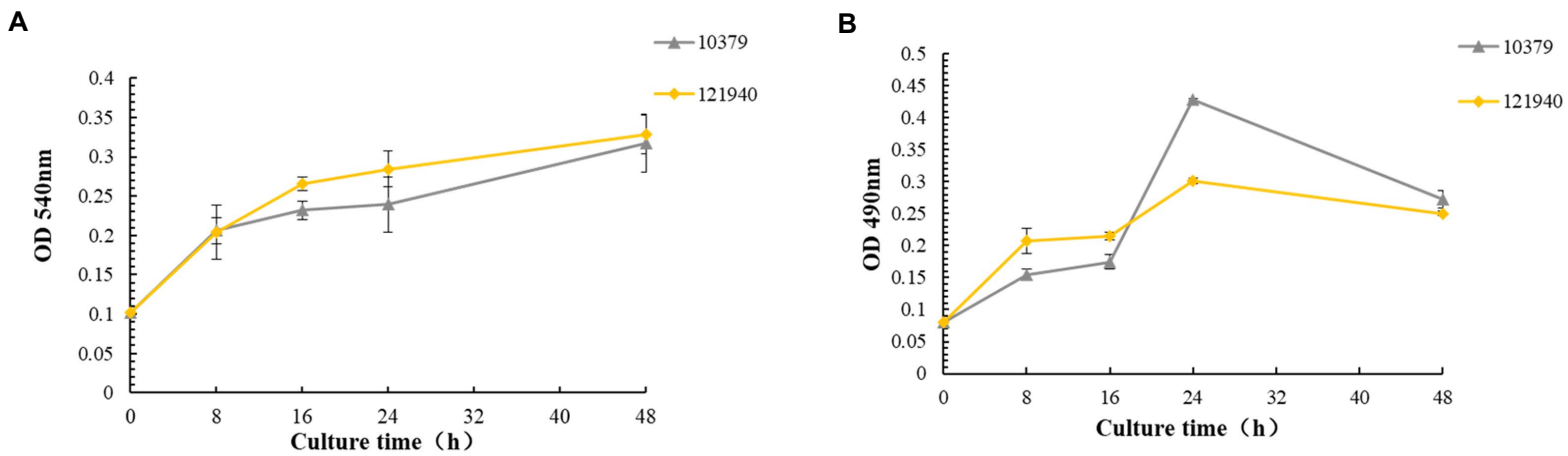
The biomass content and biofilm viability of *Staphylococcus aureus* isolates 10379 and 121940. **(A)** The biomass of two *S. aureus* isolates incubated for 48 h detected using CV method. **(B)** The Viability of two *S. aureus* isolates biofilm incubated for 48 h detected using XTT method.

The biomass content of *S. aureus* strains 10379 and 121940 was quantified at 5-time points and the growth curve of biomass content 0–48 h was plotted, as shown in Figure 1A. Also, biofilm viability of two *S. aureus* isolates was detected 0–48 h with time, and the result was shown in Figure 1B. The results indicated that both strains showed the ability to form biofilm on the solid surface and that the biomass and biofilm viability increased with the increase of incubation time, but the growth trend of biomass and biofilm viability was not entirely consistent. The biomass of both strains increased continuously from 0 to 48 h, with the biomass content reaching a maximum at 48 h, Figure 1A. However, for biofilm viability, there was a gradual increase in biofilm viability from 0 to 24 h with increasing incubation time. But at 24–48 h the biofilm viability of the strains showed a decreasing trend, Figure 1B.

The reason might be that from 0 to 24 h the two strains of *S. aureus* were in a nutrient-rich environment so that *S. aureus* proliferated rapidly and also secreted metabolites through metabolic activity. This resulted in a rapid increase in the total biomass from 0 to 24 h. From 24 to 48 h, the nutrient content of the environment gradually decreased, affecting the proliferation and metabolic activity of the bacteria, resulting in a slower increase in the total biomass (O'Toole et al., 2000). The decrease in biofilm viability from 24 to 48 h may be due to a decrease in live bacteria number or a slowdown in their metabolism, which may be associated with the exodus of the biofilm (Moormeier and Bayles, 2017).

### 3.4. Effect of beta-lactam antibiotics on the biomass of *Staphylococcus aureus*

The MIC results of the two strains of *S. aureus* against six beta-lactam antibiotics were measured by the micro-broth dilution method and the results are shown in Table 2. According to CLSI criteria, strain 10379 showed resistance to five beta-lactam antibiotics, excluding gentamycin, while strain 121940 showed resistance to six beta-lactam antibiotics. The MICs of penicillin (PEN), ampicillin (AMP), meropenem (MEM), streptomycin (STR), kanamycin (KAN), and gentamicin (GEN) were 64, 16, 8, 128, 2048, and 0.5 μg/ml for isolate 10379 and 256, 16, 128, 1024, 128, and 64 μg/ml for isolate 121940, respectively. Ten different concentrations of six beta-lactam antibiotics were used in subsequent experiments on the effects of biofilm formation in *S. aureus*.

**Table 2:** The MIC value of *Staphylococcus aureus* isolates 10379 and 121940.

Strains	MIC (μg/mL)					
	PEN	AMP	MEM	STR	KAN	GEN
10379	64	16	8	128	2048	0.5
121940	256	16	128	1024	128	64

**Figure 2:**
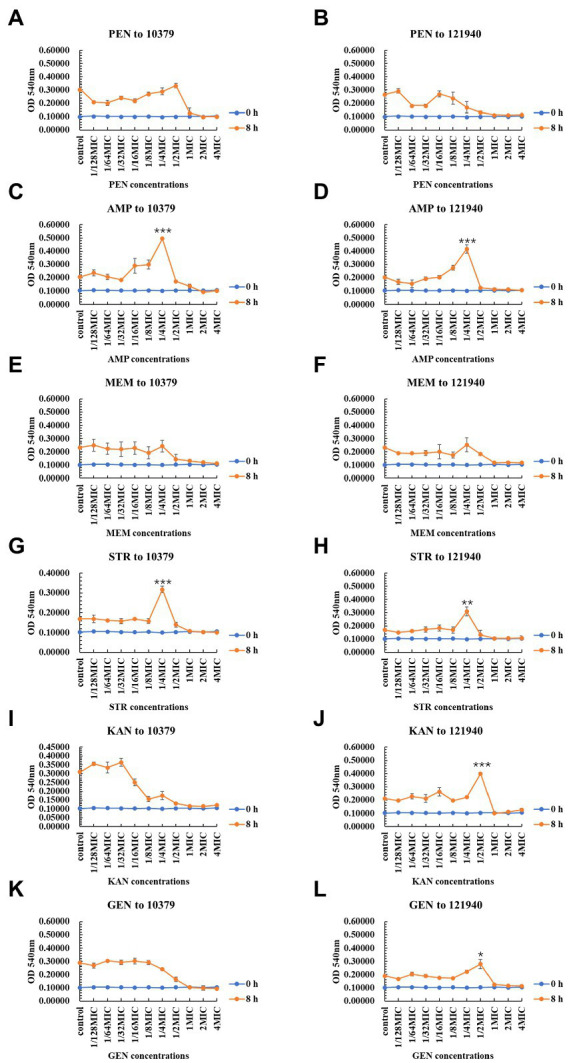
The inhibition of six beta-lactam antibiotics to the biomass of isolates 10379 and 121940 in 8 h incubation. **(A, C, E, G, I, K)** The biomass of strain *S. aureus* 10379 affected by PEN, AMP, MEM, STR, KAN, GEN, respectively. **(B, D, F, H, J, L)** The biomass of strain *S. aureus* 121940 affected by PEN, AMP, MEM, STR, KAN, GEN, respectively.

As shown in Figure 2, the six beta-lactam antibiotics inhibited biomass only at concentrations higher than MIC at 8 h incubation time. For isolate 10379, 1/4 MIC ampicillin and 1/4 MIC streptomycin promoted the formation of biomass (Figures 2C,G); for isolate 121940, 1/4 MIC ampicillin, 1/4 MIC streptomycin, 1/2 kanamycin, and 1/2 MIC gentamicin promoted the formation of biomass (Figures 2D,F,H,J,L). At 24 h incubation, 1/4 MIC and 1/2 MIC kanamycin promoted the biomass of isolates 10379 and 121940, respectively (Supplementary Figure S2). At other incubation times, low concentrations of antibiotics could also have a significant effect on the promotion of biomass formation (Supplementary Figures S1, S3). The differential effects of antibiotics on total biofilm formation in *S. aureus* strains could be associated with the specific mechanisms of the antibiotics’ action. Penicillin, ampicillin and meropenem inhibit the synthesis of cell wall peptidoglycan and they can inhibit biofilm formation only at a certain concentration threshold. Some studies have shown that sub-MICs of rifampicin has potential to stimulate *S. aureus* biofilm formation (Lima-e-Silva et al., 2017). Amit Kumar found that *S. aureus* biofilm formation is enhanced under sub-inhibitory stress of norfloxacin (Kumar and Ting, 2013).

### 3.5. Effect of beta-lactam antibiotics on the biofilm viability of *Staphylococcus aureus*

**Figure 3:**
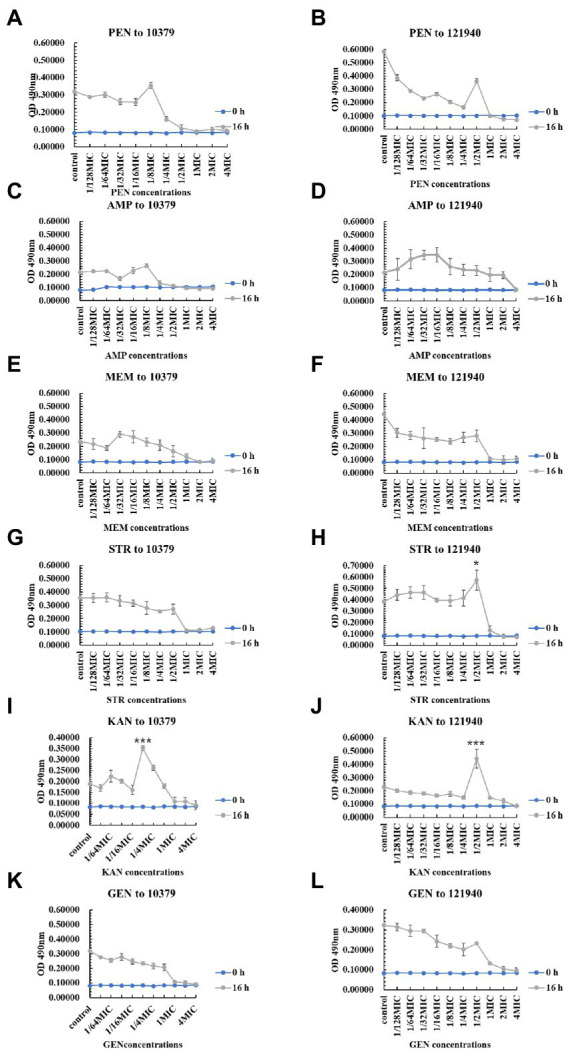
The inhibition of six beta-lactam antibiotics to the biofilm viability of isolates 10379 and 121940 in 16 h incubation. **(A, C, E, G, I, K)** The biofilm viability of strain *S. aureus* 10379 affected by PEN, AMP, MEM, STR, KAN, GEN, respectively. **(B, D, F, H, J, L)** The biofilm viability of strain *S. aureus* 121940 affected by PEN, AMP, MEM, STR, KAN, GEN, respectively.

We also studied the effect of 10 various concentrations of beta-lactam antibiotics on the 0–48 h biofilm activity of *S. aureus* 10379 and 121940. Overall, antibiotics at concentrations near the MIC were effective in inhibiting biofilm viability. However, Figure 3 showed that at 16 h of incubation, 1/8 MIC kanamycin promoted biofilm viability in isolate 10379, and 1/2 MIC streptomycin and 1/2 MIC kanamycin promoted 121940 isolate biofilm viability. In addition, 1/4 MIC kanamycin promoted the viability of strain 10379 biofilm at 24 h of culture. The biofilm viability in strain 10379 was also promoted by 1/2 MIC gentamicin at 24 h incubation (Supplementary Figures S4–S6). 1/2 MIC streptomycin was also able to promote the viability of strain 121940 biofilm at 16 h incubation (Figure 3 and Supplementary Figure S5).

During food processing, different antimicrobial process would be employed, including physical, chemical and biological methods (Liu et al., 2019, 2023). As expected, antibiotics should exhibit antimicrobial effect on microorganisms. However, low concentration of antibiotics, especially below MIC, has been occasionally reported to exhibit stimulating effect on the formation of biofilm. Kevin D. Mlynek and colleagues found that sub-inhibitory concentration of amoxicillin induce biofilm formation of *S. aureus* USA300 in static or flowing situations which may related to the production of eDNA (extracellular DNA) (Mlynek et al., 2016). Low-dose beta-lactam antibiotics lead eDNA release, significant autoaggregation, and formation of biofilm in *S. aureus.* These results are associated with the lysis of cells and the release of DNA into the surrounding environment (Kaplan et al., 2012). Beta-lactam antibiotics preferentially promote the biofilm formation of MRSA, for example, Ng et al. (2014) found that 8 *β*-lactam antibiotics induced LAC biofilms, but non-*β*-lactam antibiotics did not.

In this study, we had employed CV and XTT to separately determine different features as biomass and viability of biofilm formed by *S. aureus*, instead of using CFU as in many studies. CFU is the most commonly used method for microbiologists, however, during biofilm formation, microbial cells within biofilm exhibited diversity in viability, and a large number of cells have formed VBNC (Ayrapetyan et al., 2015a,b, 2018). VBNC formation has also been previous reported to be responsible for food poisoning and spoilage (Deng et al., 2015a; Liu et al., 2016b,c; Xu et al., 2022). Despite incapable of growing on medium plates, VBNC cells can solely express toxins or produce spoilage substances (Liu et al., 2017d; Zhou et al., 2020; Jiang et al., 2021; Ou et al., 2021). Biofilm and VBNC formation are both significant concerns in food safety, and their formation is also closely relevant to the incubation conditions. Previously, condition mimicking food processing have been used to inhibit biofilm or VBNC formation (Liu et al., 2018a, 2019; Li et al., 2020a,b,c; Wei et al., 2020). Initially, antibiotics have also been considered to be an agent employed for biofilm or VBNC formation, and generally speaking, antibiotics have an inhibitory effect on the growth of microorganisms and thus should inhibit the formation of biofilms. Nevertheless, the results of the experiment in this section show that some beta-lactam antibiotics were able to promote the growth of biofilms at sub-inhibitory concentrations.

## 4. Conclusion

According to PCR tests on the initial biofilm adhesion gene *atl*, the intercellular polysaccharide adhesin regulator gene *ica* manipulator, the aggregation protein regulator gene *aap*, and the mature biofilm regulator gene *agr* in strains 10379 and 121940, strain 10379 carried the *icaA* and *icaBC* genes, while strain 121940 carried the *icaA*, *icaD*, *icaBC*, *alt*, and *agr* genes. The results of multiplex PCR showed that SCC*mec* genotypes of both *S. aureus* 10379 and 121940 both *S. aureus* are type IV. Multilocus sequence typing of staphylococci and amplification and sequencing of seven housekeeping genes showed that strain 10379 was type ST45 and strain 121940 was type ST546. Although the two strains of *S. aureus* used in this study differed considerably in genotype, both strains showed resistance to penicillin, ampicillin, meropenem, streptomycin, and kanamycin. Beta-lactam antibiotics at concentrations above the MIC have an inhibitory effect on biofilm formation and can affect biofilm viability and biomass. At certain sub-inhibitory concentrations, ampicillin (1/4 MIC), kanamycin (1/2 MIC), and streptomycin (1/4 MIC) can promote biomass accumulation, and 1/2 or 1/4 MIC penicillin and kanamycin can increase biofilm viability.

## Data availability statement

The original contributions presented in the study are included in the article/Supplementary materials, further inquiries can be directed to the corresponding author/s.

## Author contributions

JL: writing – original draft and data curation. TH: resources and conceptualization. YM: methodology and supervision. XL: supervision and review and editing. All authors contributed to the article and approved the submitted version.

## Funding

This work was supported by the Projects of Enterprise Sci-tech Commissoner of Guangdong (No. GDKTP2021036400), the Projects of Science and Technology of Yunfu (No. 2022010220), and the 111 Project (B17018).

## Conflict of interest

The authors declare that the research was conducted in the absence of any commercial or financial relationships that could be construed as a potential conflict of interest.

The reviewer YL declared a shared affiliation with the author YM to the handling editor at the time of review.

## Publisher’s note

All claims expressed in this article are solely those of the authors and do not necessarily represent those of their affiliated organizations, or those of the publisher, the editors and the reviewers. Any product that may be evaluated in this article, or claim that may be made by its manufacturer, is not guaranteed or endorsed by the publisher.

## Supplementary material

The Supplementary material for this article can be found online at: https://www.frontiersin.org/articles/10.3389/fmicb.2023.1139753/full#supplementary-material
