# Supplementary material for: Biofilm formation of two genetically diverse Staphylococcus aureus isolates under beta-lactam antibiotics
Source: Front Microbiol. 2023 Mar 6;14:1139753. doi: 10.3389/fmicb.2023.1139753 (PMC10025342; doi:10.3389/fmicb.2023.1139753)
Supplement: Supplementary file 1 [file Data_Sheet_1.docx]

**Supplementary Materials**

Table S1 Primers used in this study

| The name of primer | Sequence（5’-3’） | Gene | The length of PCR production (bp) |  |
| --- | --- | --- | --- | --- |
|  |  |  |  |  |
| A1 | TCTCTTGCAGGAGCAATCAA | *icaA* | 188 |  |
| A2 | TCAGGCACTAACATCCAGCA |  |  |  |
| D1 | ATGGTCAAGCCCAGACAGAG | *icaD* | 198 |  |
| D2 | CGTGTTTTCAACATTTAATGCAA |  |  |  |
| B1 | ATGGTCAAGCCCAGACAGAG | *icaBC* | 1188 |  |
| B2 | GCACGTAAATATACGAGTTA |  |  |  |
| G1 | GTGCCATGGGAAATCACTCCTTCC | *agr* | 976 |  |
| G2 | TGGTACCTCAACTTCATCCATTATG |  |  |  |
| T1 | ACACCACGATTAGCAGAC | *atl* | 432 |  |
| T2 | AGCTCCGACAGATTACTT |  |  |  |
| P1 | GAAATGACTGAA CGTCCGAT | *aap* | 465 |  |
| P2 | GCGATCAATGTTACCGTAGT |  |  |  |
| C1 | GATGAGTGCTAAGTGTTAGG | *16S rRNA* | 542 |  |
| C2 | TCTACGATTACTAGCGATTC |  |  |  |
| F1 | AAAGCTTGCTGAAGGTTATG | *femA* | 823 |  |
| F2 | TTCTTCTTGTAGACGTTTAC |  |  |  |
| M1 | GGCATCGTTCCAAAGAATGT | *mecA* | 374 |  |
| M2 | CCATCTTCATGTTGGAGCTTT |  |  |  |


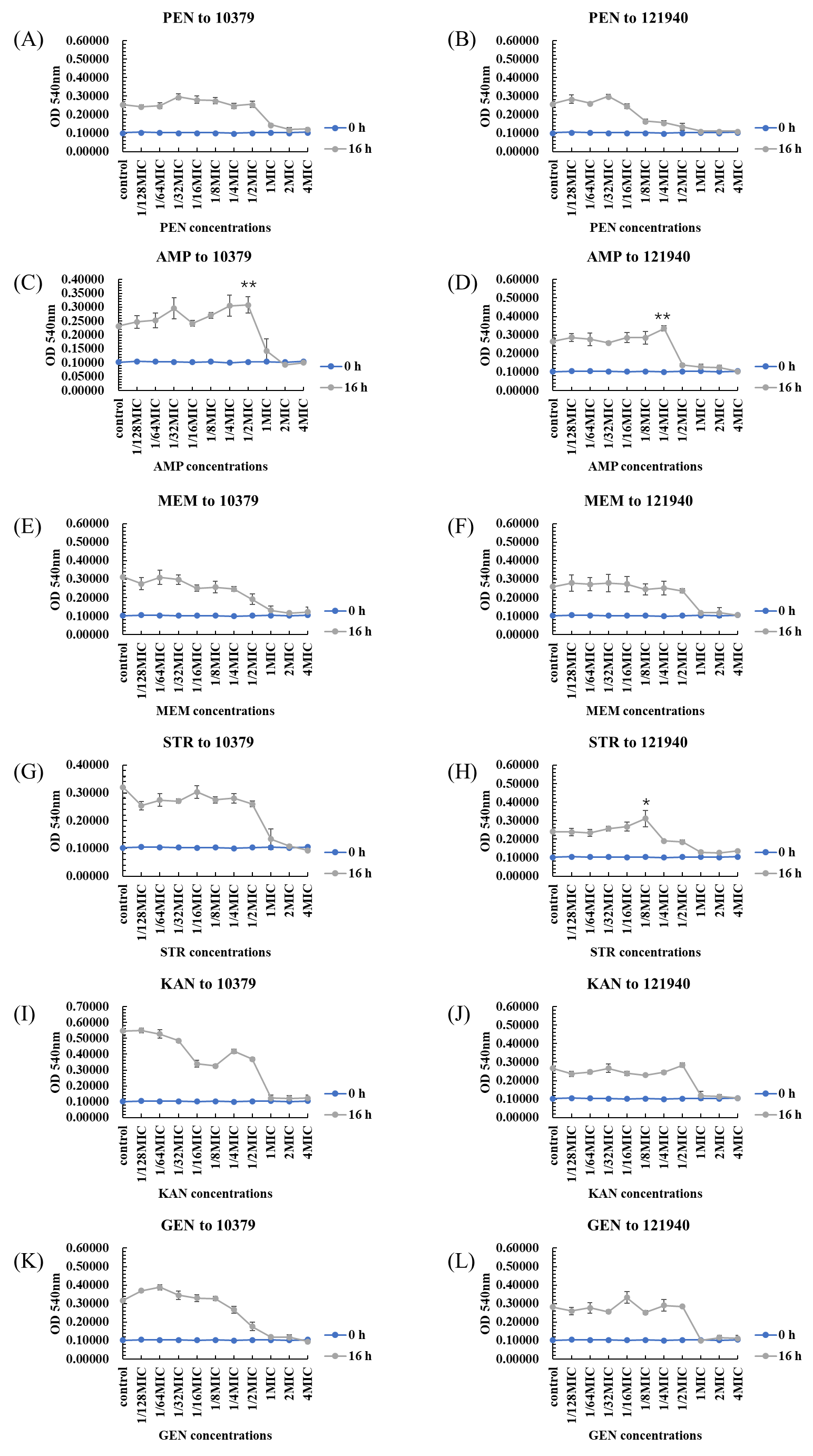


Figure S1. The inhibition of six beta-lactam antibiotics to the biomass of 10379 and 121940 in 16 h incubation.


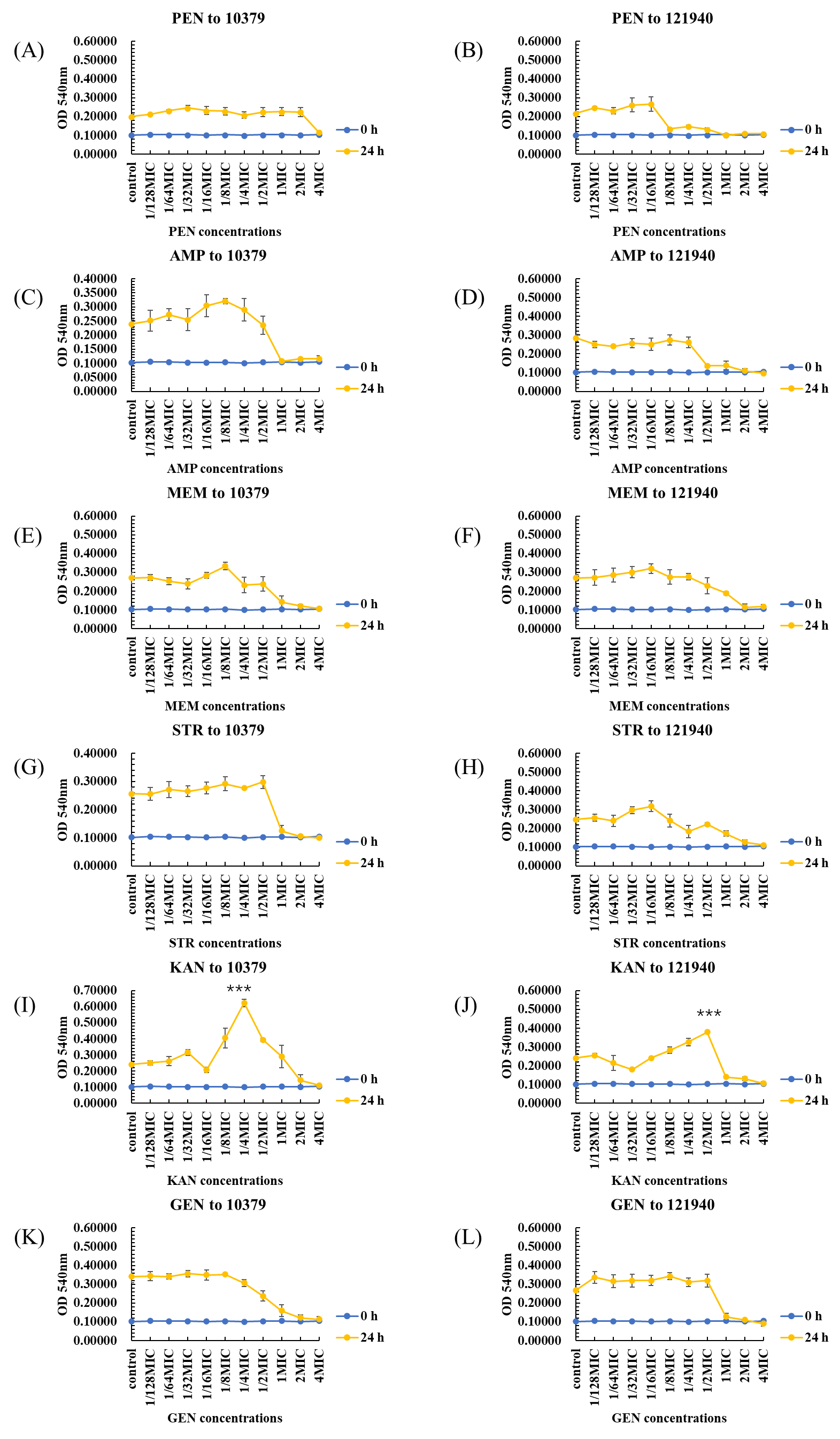


Figure S2. The inhibition of six beta-lactam antibiotics to the biomass of 10379 and 121940 in 24 h incubation.


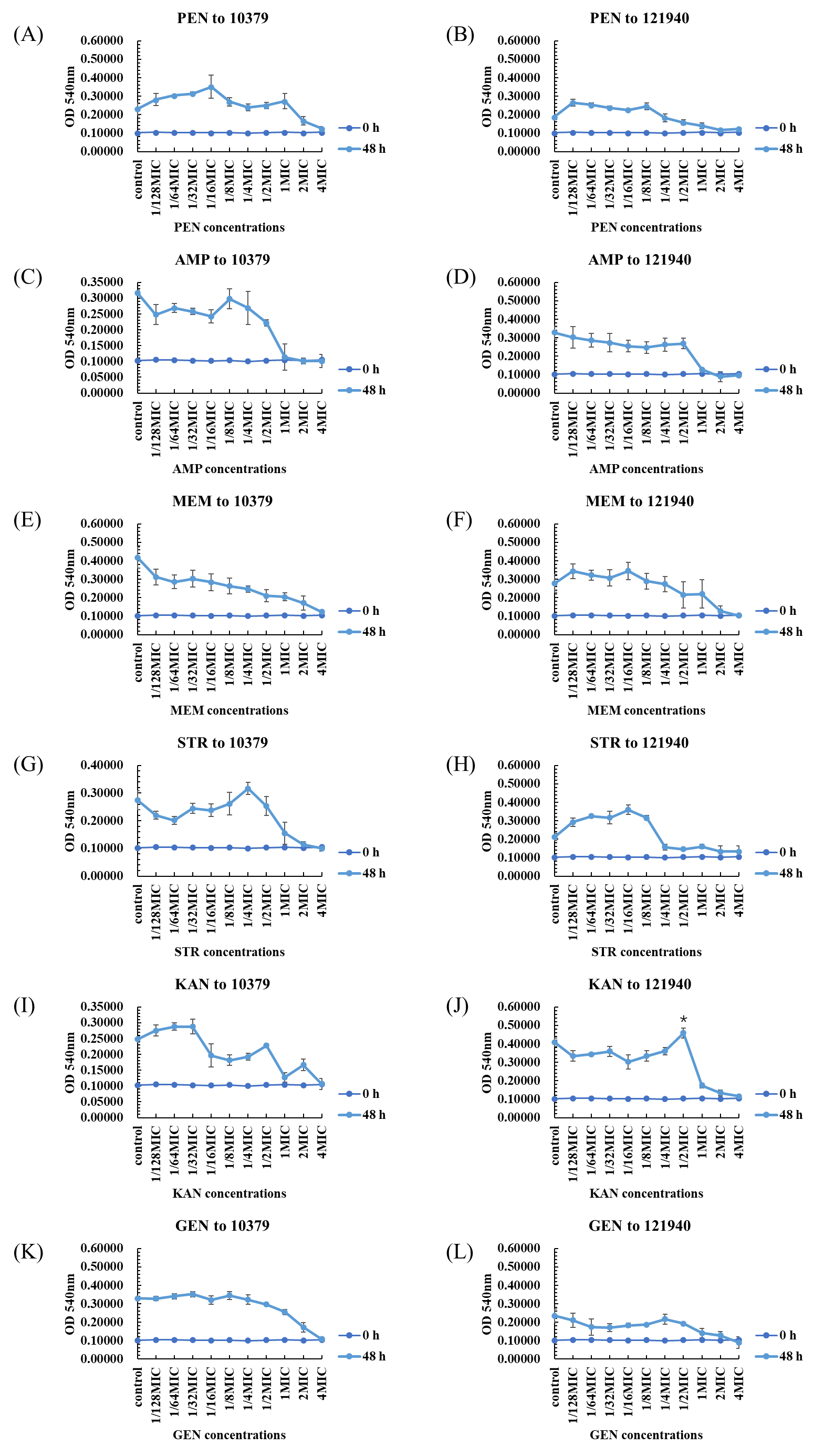


Figure S3. The inhibition of six beta-lactam antibiotics to the biomass of 10379 and 121940 in 48 h incubation.


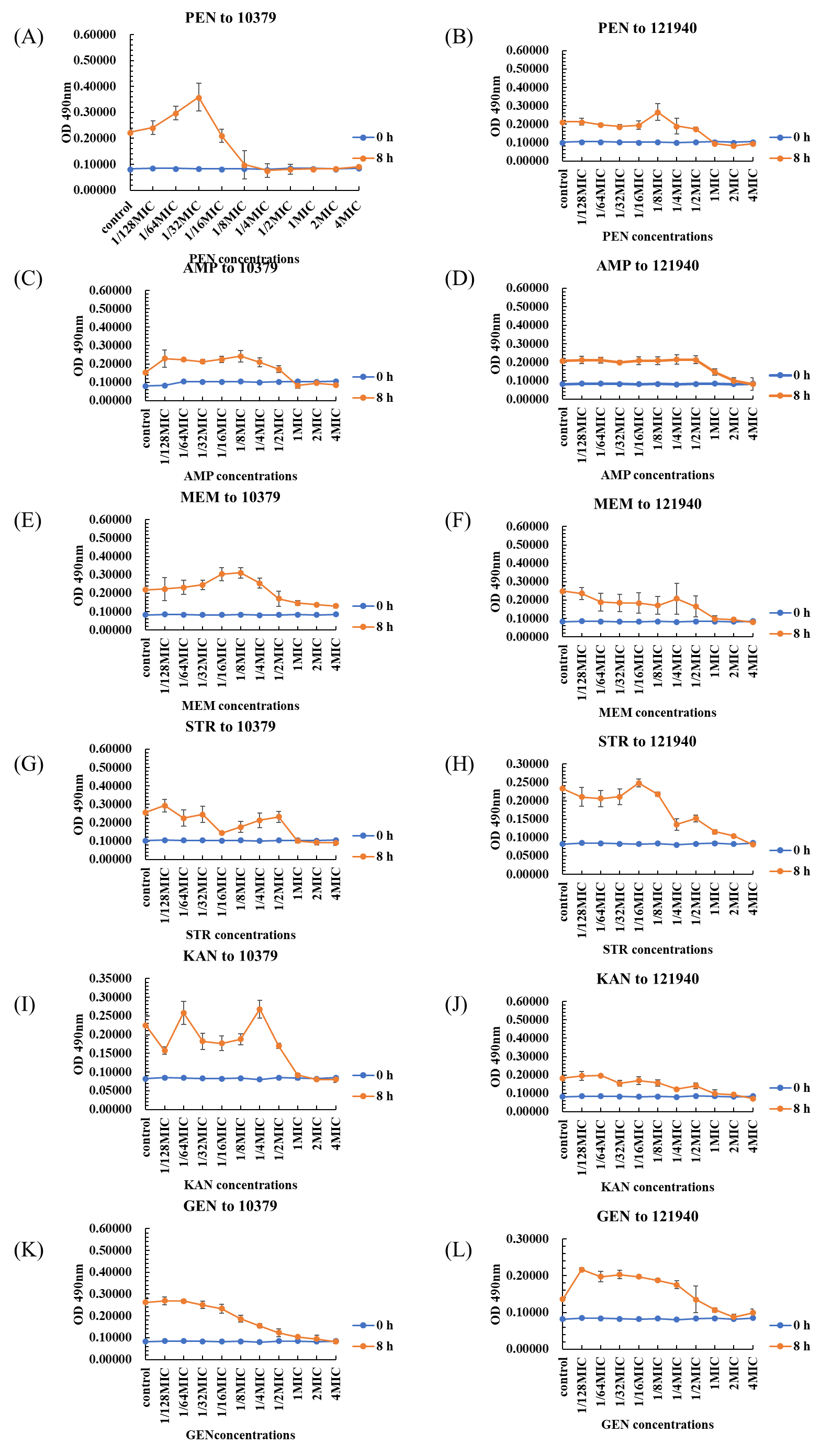


Figure S4. The inhibition of six beta-lactam antibiotics to the biofilm viability of 10379 and 121940 in 8 h incubation.


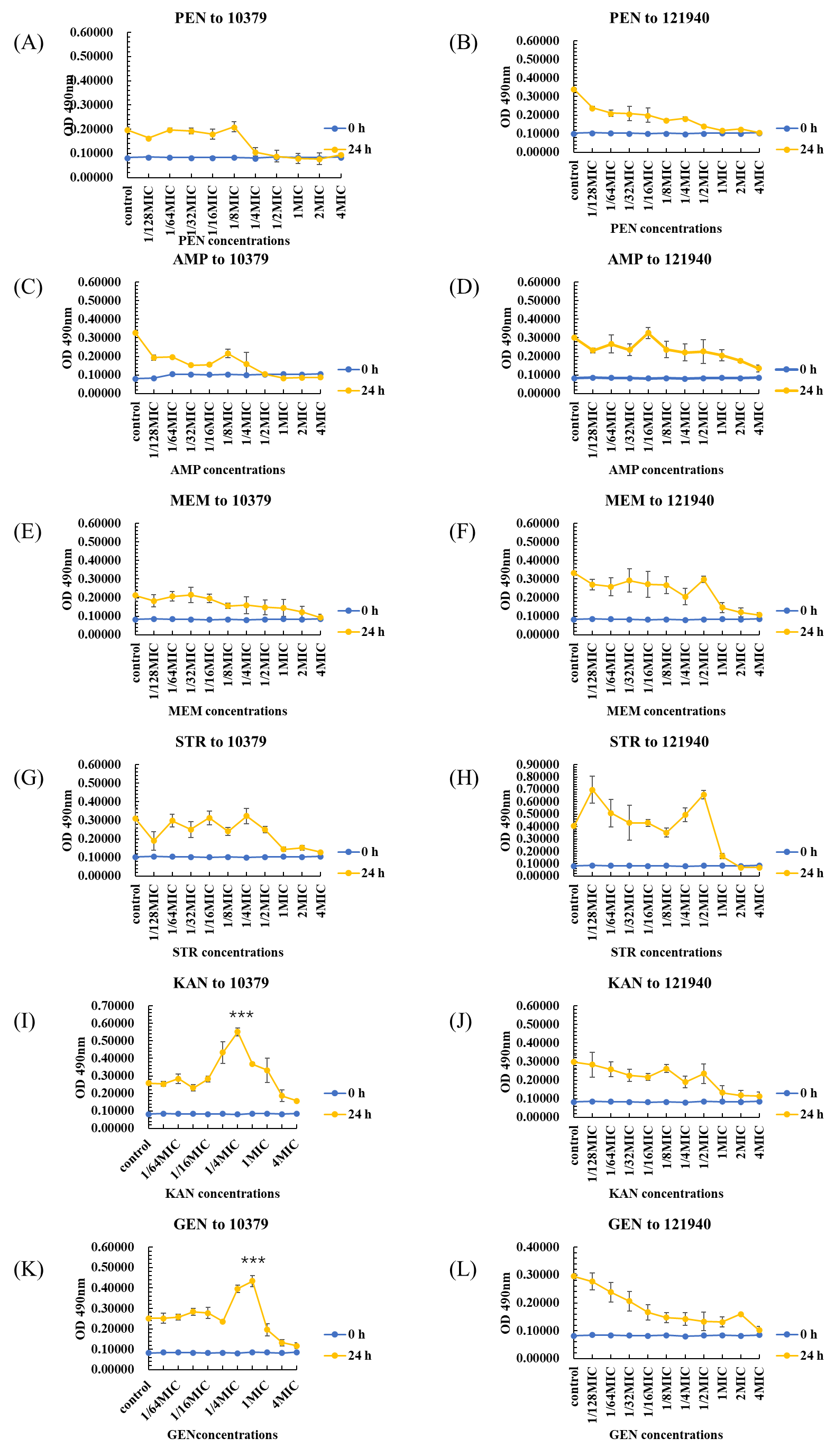


Figure S5. The inhibition of six beta-lactam antibiotics to the biofilm viability of 10379 and 121940 in 24 h incubation.


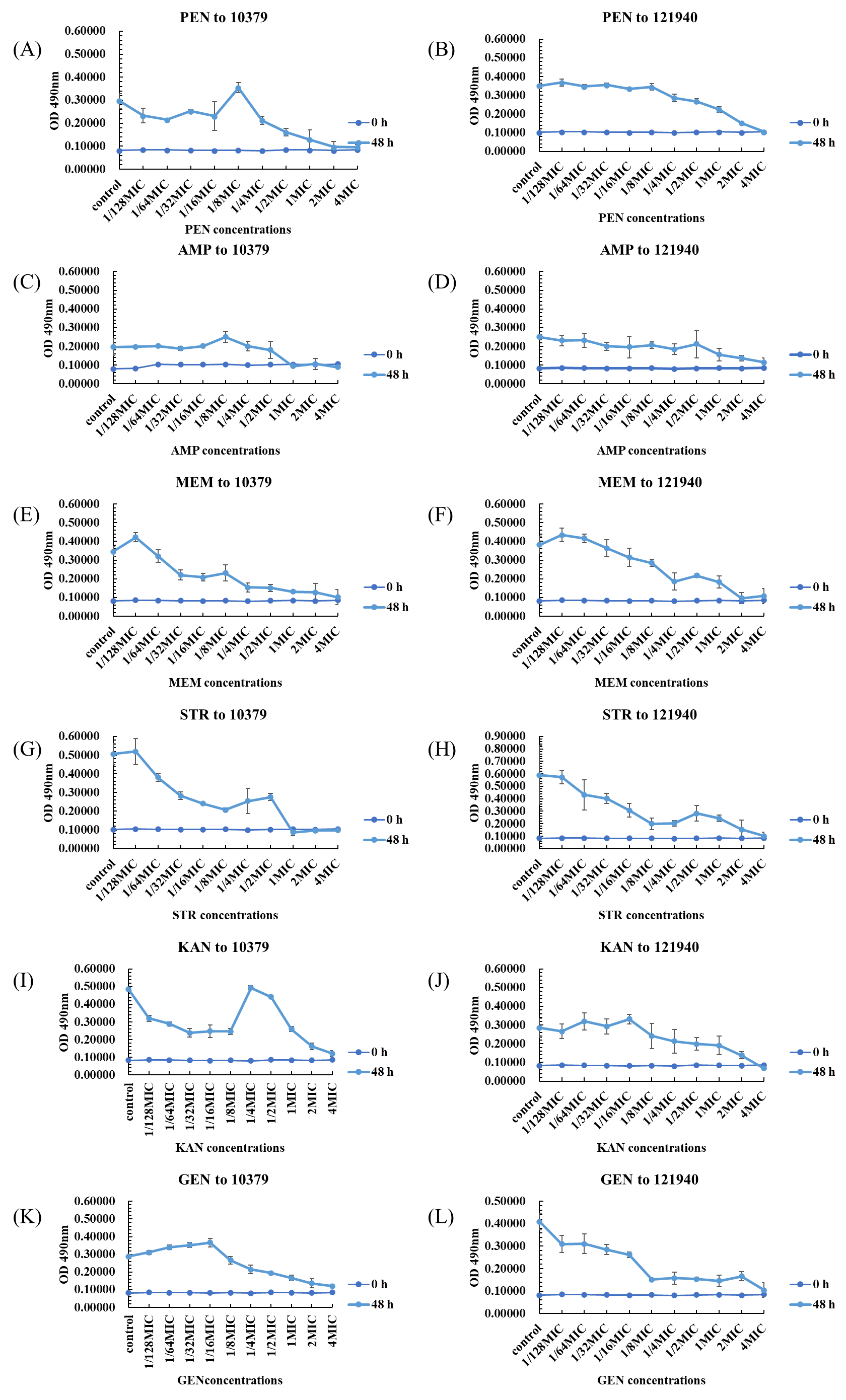


Figure S6. The inhibition of six beta-lactam antibiotics to the biofilm viability of 10379 and 121940 in 48 h incubation.
